# Heterodigital flaps for severe pulp defects

**DOI:** 10.1186/1753-6561-9-S3-A60

**Published:** 2015-05-19

**Authors:** Roberto Adani

**Affiliations:** 1 Department of Hand Surgery, Azienda Ospedaliera Universitaria Integrata Verona, Verona, 37134, Italy

## 

The forerunner of the heterodigital flaps was the standard cross-finger flap [1]. For a long time it was considered a random pattern regional flap, which employed the dorsal skin of an adjacent finger to provide soft tissue coverage for a volar defect including pulp defect. The conventional cross-finger flap provides stable padding, but sensory recovery is prolonged and often does not restore tactile gnosis. To improve the recovery of the sensibility, this flap has been modified by including one of the dorsal digital nerves to be sutured to the proper digital nerve of the injured finger [2]. This technique was even further improved including both dorsal digital nerves with the aim of achieving better sensory fiber imput [3,4]. Nowadays the cross-finger flap is considered an axial flap based on the dorsal branch artery from the radial or ulnar digital artery at the base of the middle phalanx and its design has been refined by narrowing the base [4]. The major advantage is that the proper digital artery is not sacrificed as in the “C-ring cross-finger flap” that is an axial pattern flap based either distally or proximally on a digital vascular bundle [5]. The main disadvantages of all these techniques are that they are two-stage procedures and require finger immobilisation for two to three weeks. A visible scar is also left on the donor site especially when a split thickness skin grafting is used in white people [6]. Further modifications of the cross-finger flap are reported in literature to reconstruct the dorsal aspect of the fingers [7-9].

In 1956, Littler [10] described an island flap raised from the ulnar aspect of the middle or ring finger based on the ulnar digital neurovascular bundle in order to reconstruct soft tissue defects to the thumb pulp; this is really the first described heterodigital flap harvested for pulp resurfacing. This flap was then modified including only the digital artery and the venae comitantes in the pedicle, without the digital nerve in order to minimize the sensory loss to the donor digit [11]. This “heterodigital arterialized flap” transferred from the lateral surface of a nearby digit beneath a subcutaneous bridge might be used to resurface difficult finger wounds and to cover pulp defect in the fingers. To reduce donor site morbidity and to achieve a better sensory recovery some authors have proposed to harvest the donor skin from the lateral side of the middle phalanx with the vascular pedicle located on the palmar side of the flap [12,13]. In this way the grafted donor area is aesthetically more acceptable and scar contracture can be prevented because the defect left is over the midlateral line and not extended over the palmar aspect of the finger. With this technical variation an innervated flap can be harvested; the dorsal branch of the digital nerve is constantly raising from the main nerve at this level and can easily be used to innervate the flap [14] with acceptable sensory recovery. In cases of large skin digital pulp defects of the middle finger, it may be difficult to resurface the pulp without extending the distal incision of the flap over the DIPJ crease on the donor finger since we believe that the distal pulp of the donor finger should never be harvested as part of the heterodigital flap. The solution to this may be represented by the heterodigital island flap with reverse flow vascularisation. Martin in 1994 [15] introduced the idea of harvesting a flap distally on a branch of a Y-like vascular bifurcation, and turning the Y into a V. This concept was subsequently used in hand surgery for dorsal finger reconstruction employing the dorsal digital network [16-19].

On the basis of this concept we developed a heterodigital island flap, which can be considered as a reverse- flow flap [20-22]. With this flap the pedicle is isolated up to its bifurcation in the palm and the common digital artery between the injured finger and the flap-donor finger is transacted just before its bifurcation. The Y-like vascular bifurcation then turns into a V shape and the two converging branches of the digital arteries can be now mobilized as a continuous vascular pedicle for the flap; this procedure considerably increases the length of the flap pedicle. Finally the vascularization is supplied by a “reverse-flow” system through the proximal transverse digital palmar arch of the injured finger (if the middle transverse palmar arch has been damaged) so the flap can reach the defect. The recipient digital nerve of the defect (usually the radial side) is dissected and a microneurorraphy is done to the sensory nerve of the flap. The donor defect is covered with a full thickness skin graft. At the end of the procedure, an aluminium splint is applied with the MP and IP joints in slight flexion: mobilization is started after 15 days.
